# Exploration of the Use of New Psychoactive Substances by Individuals in Treatment for Substance Misuse in the UK

**DOI:** 10.3390/brainsci8040058

**Published:** 2018-03-30

**Authors:** Rosalind Gittins, Amira Guirguis, Fabrizio Schifano, Ian Maidment

**Affiliations:** 1 Addaction, 67-69 Cowcross St., London EC1M 6PU, UK; 2 Psychopharmacology, Drug Misuse and Novel Psychoactive Substances Research Unit, University of Hertfordshire, Hatfield AL10 9AB, UK; f.schifano@herts.ac.uk; 3 Pharmacy Department, School of Life and Health Sciences, Aston University, Birmingham B4 7ET, UK; i.maidment@aston.ac.uk

**Keywords:** new psychoactive substances, substance use, cannabinoids, stimulants, substance misuse services, substance use treatment, psychosocial interventions, harm reduction

## Abstract

Substance misuse services need to meet the growing demand and needs of individuals using new psychoactive substances (NPS). A review of the literature identified a paucity of research regarding NPS use by these individuals and UK guidelines outline the need for locally tailored strategies. The purpose of this qualitative study was to identify and explore key themes in relation to the use of NPS by individuals receiving community treatment for their substance use. Electronic records identified demographics and semi-structured interviews were undertaken. A thematic analysis of transcripts identified a variety of substance use histories; 50% were prescribed opiate substitutes and 25% used NPS as a primary substance. All were males, age range 26–59 years (SD = 9), who predominantly smoked cannabinoids and snorted/injected stimulant NPS. The type of NPS used was determined by affordability, availability, side-effect profile and desired effects (physical and psychological: 25% reported weight loss as motivation for their use). Poly-pharmacy, supplementation and displacement of other drugs were prevalent. In conclusion, NPS use and associated experiences vary widely among people receiving substance use treatment. Development of effective recovery pathways should be tailored to individuals, and include harm reduction strategies, psychosocial interventions, and effective signposting. Services should be vigilant for NPS use, “on top” use and diversion of prescriptions.

## 1. Introduction

“New Psychoactive Substances” (NPS) is “a generic term for … substances produced to mimic the effects of traditional illicit drugs” [1]. Formerly known as “legal highs”, NPS have dramatically changed the UK drug scene and introduced a new challenge for healthcare professionals (HCPs) [2]. The internet and the media may have had a significant impact on the proliferation of this market. A study by Bright et al. (2013) showed that the media played a significant role in increasing the public’s awareness of new NPS, which sparked curiosity and increased use. This, in turn, created a media “moral panic”, resulting in legislative reactions, which led to the emergence of more harmful substances [3]. At a global level, NPS are unregulated products with unpredictable effects and clandestine chemists continuously and rapidly produce newly modified compounds [4]. The analysis of NPS products have found controlled substances, mixtures of active substances and different constituents even from the same supplier and using the same “brand” name [1,4,5]. People who use NPS present with unpredictable adverse effects, creating a dilemma for HCPs [6]. Previously, NPS were often labelled as “not for human consumption”, so that under the Medicines Act 1968, manufacturers of NPS were not legally required to list their ingredients or determine their safety [1,4,7,8]. The UK Government subsequently introduced the Psychoactive Substances Act (UK PSA) in May 2016, to prohibit NPS sale, supply, production, possession with intention to supply or possession in a custodial institution, with an aim to reduce use [9,10].

Owing to their continuous emergence and sheer numbers (increased from 166 substances in 2009 to over 740 in 2016) [11], NPS are categorized based on their chemical class [12], legal status [13] or chemical structure [14]. However, in clinical practice, NPS are more commonly considered in the context of their pharmacological effects and the substances they have been designed to replicate. Public Health England (PHE) [15] therefore categorizes NPS as: sedating, stimulating, hallucinogenic, cannabinoids, dissociative and “other” (not otherwise specified). Consequently, these PHE categories [15] are used by substance misuse services (SMSs) to record information on the National Drug Treatment Monitoring System (NDTMS) [16], which enables the monitoring of people receiving treatment and national comparisons.

There are a variety of guidelines available to support HCPs working in mental health and SMSs, such as those produced by the Department of Health [17] and the Novel Psychoactive Treatment UK Network (NEPTUNE) [4]; however, none cover specific details for all NPS categories. Due to their “novelty”, there is a lack of clinical data to support an evidence-based approach to the management of individuals who use NPS [1]. Recent studies have shown that HCP baseline knowledge of NPS is poor and that they are less confident in managing acute toxicities related to the use of NPS compared with traditional illicit drugs [18,19]. Reports by both the Care Quality Commission and the HM Inspectorate of Probation showed that SMSs did not offer NPS-specific interventions and that people who use NPS poorly engaged [20]. Similarly, in Europe, HCPs in Italy within addiction, psychiatry, pediatrics and A&E services also reported a lack of knowledge and confidence with regards to NPS. They also affirmed that no questions are asked when taking drug histories during admission [2].

To understand the motivation for using NPS by individuals registered within SMSs, a detailed electronic search of NHS evidence was undertaken using the Allied and Complementary Medicine (AMED), Excerpta Medica Database (EMBASE), Health Management Information Consortium (HMIC), British Nursing Index (BNI), Cumulative Index to Nursing and Allied Health Literature (CINAHL), Medline, PsycInfo and Health Business Elite databases. This identified that the rapid proliferation of NPS use may be changing drug taking habits (by displacing or supplementing pre-existing drug repertoires), and may be affected by availability, price, purity and legal status [1,7,21,22]. In Ireland, in depth interviews found people switching from illicit substances to “legal” NPS, due to perceptions regarding improved effects, safety profile and overall higher quality for a lower price; however, the individuals were aged 18–33 years [23] and adult SMSs frequently treat people who are older than this. Another group of Irish interviewees were found to transition from nasal administration to injecting and binged excessively for long durations on stimulant NPS, but after they became controlled substances, their use reduced following headshop closures, increased prices and concerns about contamination [24]. Qualitative case reports of people using stimulant NPS in the UK, prior to the introduction of the UK PSA 2016, suggested their euphoric effects may lead to “more persistent patterns of drug use” due to their perceived legal status and poor quality of their illicit equivalents [21]. The analysis of cryptomarkets such as Alpha Bay, Valhalla, Agora and Evolution Market Place has also allowed the exploration of views and perspectives of vendors and customers on a large scale [25,26,27,28]. A study by Van Hout and Hearne (2017) has identified that cryptomarket customers prefer the sequential and concurrent use of psychedelics as well as NPSs, in particular, GABA (γ-aminobutyric acid)-activating NPSs [29]. The study showed that research of these market place forums can provide an insight on novel trends of NPS use. Similarly, in their study, Bright et al. (2013) utilized Google Trends as a data collection tool of NPS-specific news and employed it to generate media links related to “Kronic”—a Canadian brand known to contain cannabis. Interviewees stated that they are mainly motivated by the poor detectability of this substance at workplaces, which outweigh the unknown harms associated with this substance [3].

In contrast, a field study of people attending “gay dance clubs” in London, who may be considered early adopters of new substances, found they were not deterred by changes in legislation and that stimulant NPS were more popular than any other substance [30]. The same survey found their addition to existing drug repertoires to supplement more established “club drugs” (e.g., ecstasy and cocaine), rather than replacing or displacing them [30]; however, use was affected by availability and purity [22]. A survey of experienced users in Holland also found that people who preferred stimulant NPS were undeterred by legal status and did not displace other illicit substances [31].

Perhaps the reasons for these conflicting findings is that individuals may not always be open or honest about their substance use behaviors when questioned, and NPS packaging may not always contain the expected substance [1,5,32]. Therefore, some novel approaches have been utilized, such as surveying websites and online forums such as Facebook and Twitter [29,33]. The former study found that NPS were used for pleasure, out of curiosity, alongside or as an alternative to other substances [33], whereas, the latter highlighted the diverted use of prescription medicines and poly-drug use due to “high level of social media engagement” [29]; however this methodology requires individuals to have internet access, which may not always be possible for or desired by people who have problems with substances, for example, if they are leading particularly chaotic lives or are homeless. Analyzing samples in isolation also does not allow for exploration of confounding factors or perhaps more importantly, discussion with individuals regarding their reasons for use, experiences with them or any treatment needs.

Historically, SMSs have focused on provision of services for people using crack-cocaine and opiates because these substances previously dominated the UK drug market [15]. Currently, people using NPS are increasingly presenting to SMSs with physical and psychological problems, including dependency [4,8,21]. Researchers working at the Camden and Islington NHS Foundation Trust studied 442 people who were engaging in substance use admitted to a mental health unit. The sample comprised 58 people who use NPS among which, 32 initially presented to A&E, 29 involved the police, 30 were sectioned under the Mental Health Act, and 46 presented with violence before and during admission; most of them were poly-substance users [34]. SMSs therefore need to adapt and change to meet the growing demand from people using NPS [8,35]. Once engaged, limited treatment data suggests that individuals often respond well, with high rates of successful treatment completion when compared to other substances [15]; however, concerns about poor engagement of high-risk people who engage in risky NPS use (e.g., MSM (men who have sex with men)) with SMSs remain [36]. They may present elsewhere such as sexual health services; therefore, this data may not be captured by NDTMS [37]. On general adult inpatient wards of a Scottish psychiatric hospital, NPS use was found to be prevalent among 22% of young male inpatients, in particular those with drug-induced psychosis and often used with other drugs including cannabis and prescribed opiate substitutes [38]. In Hungary, a study by Kapitány-Fövény et al. (2017) also outlined stimulant NPS prevalence among individuals receiving opiate substitution therapy. The main reasons for NPS use were curiosity and practical reasons (including availability) rather than psychopharmacological preferences [39].

Further research is required into the use of NPS by those attending SMSs as highlighted in the 2014 Home Office review [1]. For example, a large Hungarian needle exchange program study found that high risk drug users switched from injecting predominantly illicit amphetamine to NPS and the study recommended further exploration of purity, price and availability [40]. In depth interviews of eleven Irish high-risk stimulant NPS injectors recommended further investigation into their adverse health effects and displacement of other drugs [24]. Some people in SMSs use non-stimulant NPS and do not always inject, so further research is required, which should include all NPS types used via different routes of administration. UK research is particularly lacking, and more is required because SMSs, legislation and illicit drug supply chains can be very different to other countries.

As described above, published research, which definitively outlines the use of NPS by individuals, who engage with specialist adult SMSs in the UK was lacking. Due to the paucity of research, this study will consequently have national and international resonance, contributing to existing knowledge of how NPS may be changing drug taking habits and consequently positively impact upon current practice.

The aim of the study was to explore NPS use by individuals receiving treatment for substance use by exploring their type and pattern of NPS use and associated positive and negative experiences.

## 2. Materials and Methods

In the South West Peninsula of England, the charities RISE (Recovery and Integration Service) in Devon (excluding Plymouth and Torbay) and Addaction in Cornwall have been commissioned to provide integrated specialist SMSs (where RISE is a subsidiary of Addaction, a national organization). RISE and Addaction work with people regardless of which substance(s) they have a problem with. They cover predominantly rural areas, where national data suggests that NPS use may be more common [1,15], although the reasons for this are poorly understood. The use of NPS by the population who engage with these services has not been investigated. This is required because, in accordance with PHE guidance [15], an improved, local understanding of NPS use is important to enable the development of an effective recovery pathway, which appropriately supports the needs of those requiring treatment. There is limited information on international, national or local recovery pathways, to which these SMSs can refer. Ethics approval was obtained from Aston University’s Life and Health Sciences Ethics Committee on the 20 January 2015 (Project Identification Code No. 726). The study was reviewed and approved by Addaction/RISE internal governance processes on 13 February 2015. Time was then taken to undertake the study and to seek approval for its publication.

### 2.1. Study Design: Methodological Orientation and Theory

This was an explorative, qualitative study using thematic analysis [41,42,43,44]. Purposive sampling [41,42], where all eligible individuals (as outlined by the inclusion and exclusion criteria below) were identified by their Recovery Co-ordinators (RC) and invited to participate in face-to-face in depth semi-structured interviews [41,42], enabled a range of relevant views to be obtained. This methodology was chosen because it is suited to exploring knowledge in poorly understood areas such as an individual’s use of NPS [41,42,43,44]. Such methodology has been shown to be successful in obtaining detailed information from individuals regarding their NPS use [42,43,44]. This study has been reviewed and approved by Addaction’s internal governance processes and Aston University’s Life and Health Sciences Research Ethics Committee.

Anonymizing results to protect confidentiality and the use of the same researcher, who has no prior knowledge of the interviewees (or ability to routinely impact on their care such as changes to their prescribed treatment) should have increased the likelihood of individuals sharing their personal views and experiences [42,43,44]. The interviews were conducted by a qualified pharmacist with many years of experience of working in mental health and substance misuse services.

### 2.2. Study Design: Participant Selection and Setting

Potential participants were contacted by their RC (who identified that the person had experience of NPS) and individuals were provided with an information sheet and consent form which provided a full explanation of the study. The person’s ability to provide informed consent [45,46] and eligibility to participate was confirmed immediately prior to the interview. The interviews took place at one of the SMSs or established partner agency sites, where the individual usually attends for their treatment reviews. The presence of non-participants such as the person’s RC was permitted if requested by the individual.

Inclusion criteria: Previous experience of using NPS and receiving community treatment (pharmacological/psychosocial) for substance use with the charities RISE in Devon or Addaction in Cornwall.

Exclusion criteria: In receipt of in-patient or prison services; known in a clinical capacity by the Researcher; presented with significant risk issues (after assessment by their RC); additional needs could not be met (for example if they required an interpreter because their first language was not English and the SMS was unable to facilitate) or lacked capacity to consent: particular care was taken if the person was thought to have a disability, mental health problem or thought to be under the influence of substances [47,48].

### 2.3. Study Design: Data Collection

Halo is the electronic record system utilized by SMSs in the South-West Peninsula of England. Quantitative data was collated from the Halo system to identify key characteristics including the participants age, sex, employment, housing status and primary substances they use (such as opiates, cannabis, stimulants and alcohol), which the system classifies in accordance with PHE [15] for the NDTMS [16].

The interview guide (Supplementary Information 1 (SI1)) was piloted to ensure it was suitable for use, and open questions were used to avoid leading the person’s responses. The piloting process involved local SMS managers and experienced HCPs reviewing the questions. The interviews were digitally recorded and fully transcribed verbatim [41]. The intention was to undertake approximately twenty interviews to achieve a degree of data saturation, where no new or relevant information is elicited; however, research suggests that as few as eleven or twelve may be required [49], and this was found to be the case in this study.

### 2.4. Data Analysis

During the transcription process any potentially identifying information was deleted and only Halo identification codes used to identify participants. Thematic analysis, which has been specifically designed for applied qualitative research that commences deductively from specified aims and objectives, was used to organize and make sense of the data using a framework approach [41,42,43,44]. Initially, a framework was developed from existing literature; this was then updated based on the new data obtained from the interviews using a stepwise approach: (a) interviews were audio-recorded and transcribed verbatim; (b) familiarization: initial recurrent themes were identified following immersion in the transcripts; (c) coding: researcher applied line-by-line codes to describe key issues, concepts and themes by which the data was examined and referenced; (d) developing an analytical framework: after coding the initial transcripts, the researcher in consultation with supervisor, developed a set of codes that could be applied to all subsequent transcripts. Codes were then grouped into categories; (e) applying the analytical framework: data was attached to the framework of codes and categories; (f) developing a framework matrix: to manage and summarize the data, it was put on to the framework to which the data related. At this stage all transcripts were actively searched for results, which contradicted the key conclusions; (g) data interpretation: the framework matrix was used to define concepts and discover associations between the themes to provide explanations for the results; and (h) the end result was a matrix of themes from each source.

## 3. Results

Twelve individuals participated in this study between July and September 2015 and their demographic particulars are summarized in Table 1. All participants were in receipt of psychological interventions, with an age range of 26–59 years (SD = 9; mean 37). Three people (all within Addaction Cornwall) were known to the Criminal Justice Team as they were subject to Court Treatment Orders (CTOs) for their substance use.

During the interviews, one participant (P_9_) was accompanied by his RC following his request. Interviews had a mean duration of 11 min 23 s (with a range of 4 min 45 s to 24 min 57 s). No individuals had additional needs, which required support during the interviews. The data was collated and manually analyzed by the same researcher. When transcripts were independently reviewed for themes, the findings were discussed, and no differences were identified.

Using thematic analysis [41,42,43,44], data from the interview transcripts were assigned to four core categories:

1. Substance use history;
2. Type and pattern of NPS use;
3. Positive experiences associated with NPS use;
4. Negative experiences associated with NPS use.

Thematic analysis [42,43,44] enabled further explorations of the data and identified sub-themes which are evidenced in Table 1, Table 2, Table 3 and Table 4, including participants’ quotations. A summary of the frequencies of the sub-themes is presented in supplementary information (SI2).

1. Substance use history

Individuals described a wide range of substance use histories and were at different stages in their recovery journeys; some described more complex histories and most used a variety of substances. There were varying perceptions of the severity of their substance use and some described ongoing entrenched behaviors:

“I’d been in accidents and the doctors had had me on codeine and dihydrocodeine and slow release morphine and stuff … I just gradually got used to it over the years and years and years and because I didn’t have a regular doctor, I was seeing a different doctor each time who just kept giving me scripts … My body got used to it and I just started taking more and more … I’d just split up with my wife, and sort of going through a bit of a dodgy patch” **_P1_**

“I’m a recovering alcoholic … Anything that would go up my nose was going” **_P2_**

“Fertiliser–from off the farm … I inject heroin … tried every drug in the alphabet … got a crack addiction … started having counselling … gotta get my life sorted … going to rehab … Childhood–stepdad beat me up …” **_P3_**

“I fell in with the wrong crowd … I lost both my parents in a car crash so sort of went downhill and then sort of started using heroin” **_P4_**

“Started taking mind altering substances when I was 16 … these 20 years … changing my mental state on a daily basis … good help from a drug worker … got a job … more stable … My childhood trauma is what they think kind of led to my habitual use of drugs” **_P5_**

“I can’t put drugs away … just work my way through them to the bitter end” **_P6_**

“I don’t really know why I take them anymore. I think it’s just a habit” **_P7_**

“I used to do it [speed-balling] quite a lot … since I was a kid … once a month or so, I might have a drink but nothing to excess … never more than a bottle of whiskey” **_P11_**

2. Type and pattern of NPS use

**Table 2:** Type and pattern of NPS use. A list of all the themes and sub-themes, with respective quotes by participants.

Participant	Quote	Theme	Sub-Theme
P_1_	*Plant food … Snorting it … Just a couple of times … it wasn’t really something I got into heavily or used a lot … just a couple of times … A mate of mine had it at his house*	NPS type	“Other” (not otherwise specified) only
		Source	Friend only
		Frequency	Occasional
		Route of administration	Nasal only
P_2_	*Sparkle … exodus … I’m injecting [i/v] … back of my hands, my arms … I’m now snorting instead … I’m surprised they [veins] haven’t collapsed yet to be fair … I was taught by the best … Sat down for an afternoon … talked process and cleanliness [injecting technique] … About three or four [times a day] it used to be five or six … I’m using another to come down on [synthetic cannabinoid] … a couple of tokes … at the end of the session, however many days that is-3 or 4 … I tend to get my money at the beginning of the fortnight and I’ll plan out for it. I’m a typical user … [from a headshop] It’s easier than buying, chasing a dealer*	NPS type	Stimulating and Cannabinoid
		Source	Headshop only
		Frequency	Daily (several times)
			Binging
		Route of administration	Nasal and Intra-venous injection
		Concomitant use	Cannabinoid NPS to end stimulant NPS use
		Preference	Accessibility
			Avoid dealers
		Harm reduction	Changed route of administration
			Safer injecting training
		Affordability	Budgeting
P_3_	*Mephedrone … Cherry Bomb [cannabis type] … speedy … Gocaine … smoke … snort powder … banging up … It was cheap … shops and online … you can buy it … in a kilo and sell it off in ten bags … made a lot of money … dealt loads*	NPS type	Stimulating and Cannabinoid
		Source	Headshop and Online
		Route of administration	Nasal, Smoking and Intra-venous Injection
		Affordability	Cheap
			Dealing
P_4_	*Pink Panthers and Eisenberg … represent speed … uppers … Black Mamba … I smoked them but I have been known to inject a Pink Panther … I dibbled and dabbled … it wasn’t an everyday thing, only you know once every 3 or 4 weeks … and then when I would come home, I would do a bit of heroin to go to sleep … [head] shop*	NPS type	Stimulating & Cannabinoid
		Source	Headshop only
		Frequency	Occasional
		Route of administration	Smoking and Intra-venous Injection
		Concomitant use	Opiates to end stimulant NPS use
P_5_	*[Speedy ones] the names they had for them were things like Gogaine, or Posh … Stimulants were either taken orally or snorted … if I was smoking synthetic cannabis, I would be using tobacco with it … constantly … either be becoming high on synthetic stimulants or coming down off that and using synthetic cannabis … if I’d just been paid … in one hit and then top up throughout the week … I go in there for one thing, the temptation to buy something else … My willpower was helped by a change of circumstances and that change of circumstances in that the shop’s no longer selling it*	NPS type	Stimulating and Cannabinoid
		Source	Headshop only
		Frequency	Daily (several times)
		Route of administration	Oral, Nasal and Smoking
		Concomitant use	Cannabinoids with tobacco
			Cannabinoid NPS to end stimulant NPS use
		Preference	Accessibility
		Affordability	Budgeting
P_6_	*The pot ones … Spice … Scorpion … Clockwork Orange … Pandora’s Box … Toxic Waste … Smoking it neat in a pipe … in a rolled cigarette … I found that the dosage is important … I’d do very small amounts … I was writing it down, how much I was doing, taking tiny puffs … I was on methadone at the time … I substituted it [with NPS] … At the newsagent, you were able to buy them pre-rolled and my friend who smokes pot … headshop … online from some dodgy retailer … Couldn’t get any marijuana*	NPS type	Stimulating and Cannabinoid
		Source	Headshop, Friend, Online and Other (newsagent)
		Route of administration	Smoking only
		Concomitant use	Displacement (prescribed opiates)
			Cannabinoids with tobacco
		Preference	Accessibility
		Harm reduction	Changed dose
P_7_	*Diamond Dust, Lush, Sparkle [speedy types] … Crystal … IV … In my arms … Since I’ve been doing legal highs I haven’t touched it [heroin] … legal high shop*	NPS type	Stimulating only
		Source	Headshop only
		Route of administration	Intra-venous injection only
		Concomitant use	Displacement (illicit opiates)
P_8_	*Low Rider, Cotton Candy, Strawberry Cough … cannabis types … Smoking them, putting them into joints or into a pipe … just this and tobacco … Every 20 min … buy it in bulk of tens … headshop*	NPS type	Cannabinoid only
		Source	Headshop only
		Frequency	Daily (several times)
		Route of administration	Smoking only
		Concomitant use	Cannabinoids with tobacco
		Affordability	Bulk-buying
P_9_	*Mephedrone … Devil’s Dust … an upper, a stimulant … synthetic cannabinoids … IV [groin] … smoking [cannabinoids] … No more often than once a day [cannabinoids] … About every quarter of an hour [stimulants] … obviously there were sleep breaks but it was about a 6-month period … I didn’t withdraw from opiates … it took me away from heroin … Group of friends and from a chap*	NPS type	Stimulating and Cannabinoid
		Source	“Street dealer” and Friend
		Frequency	Daily (several times)
			Binging
		Route of administration	Smoking and Intra-venous Injection
		Concomitant use	Displacement (illicit opiates)
P_10_	*The powders and the puff … Smoking it. Injecting it … snorting everything … Stopped me drinking … no withdrawals … Legal high shop … Dealers as well*	NPS type	Stimulating and Cannabinoid
		Source	“Street dealer” and Headshop
		Route of administration	Nasal, Smoking & Intra-venous Injection
		Concomitant use	Displacement (alcohol)
P_11_	*Mephedrone … Methalone. A couple of hallucinogenic ones. And the pills that they did … Hawaiian Woodrose … They’re a seed basically and they’ve got an LSA in them which is like a precursor to LSD … smoking … Usually I would just have a couple of Valium [to help come down from NPS] … Internet … It was just a sort of weekend thing … Not very regularly.*	NPS type	Stimulating, Dissociative and Hallucinogenic
		Source	Online only
		Frequency	Occasional
		Route of administration	Smoking only
		Concomitant use	Benzodiazepines to end NPS use
P_12_	*Crystal, Boom Dust … Diamond Dust mephedrone … smoke the cannabinoids … Lotus … The speedy ones … Crystal Meth ones–the uppers … opiates from Italy … I snort them … dabble them … Put it in your mouth, let it dissolve … smoking if they’re the cannabinoid ones … Putting it in with baccy, it makes it last longer … If you mix them together, you hallucinate … It’s opportunist … just down the road...just go to the shop and don’t get ripped off by the drug dealers … every day.*	NPS type	Stimulating, Cannabinoid and Sedating
		Source	Headshop only
		Frequency	Daily (several times)
		Route of administration	Oral, Nasal and Smoking
		Concomitant use	Potentiating effects
			Cannabinoids with tobacco
		Preference	Accessibility
			Avoid dealers
		Affordability	Cannabinoids with tobacco

3. Positive experiences associated with NPS use

**Table 3:** Positive experiences associated with NPS use. A list of all the themes and sub-themes, with respective quotes by participants.

Participant	Quote	Theme	Sub-Theme
P_1_	*I wasn’t thinking about the ex-wife … The buzz … like being drunk without the drink … fuzzy, happy, chilled out sort of feeling*	Psychological effects	Escapism
			Relaxation
			Happiness
			Euphoria
P_2_	*It allows me not to be me … Without them, I’m a totally different person, I would never talk you right now without it … allows me to be confident … Part of it was to lose weight … I was 17 stone when I started out on it … just 10 times better than the drink … It’s stronger than street*	Psychological effects	Escapism
			Confidence
		Physical effects	Weight loss
		Preference	Strength
			Quality
P_3_	*They keep me awake when I was out clubbing … Was I allowed to sell them? I thought I was cos it was legal*	Psychological effects	Alertness/Energy
		Preference	Legal status
P_4_	*It was about a fiver whereas you go out and buy a gram of coke and its 60 quid*	Preference	Affordability
P_5_	*Wanting to escape from reality any way, or change that reality … [stimulants] a heightened sense … very energetic … happy … [cannabinoids] lethargic, very drowsy … most of the time dozing on the sofa in front of the TV-that is what I wanted … It’s hundreds of times stronger … would last for ages … I got bored … I was drawn to try them … It’s cheaper [than marijuana] … 13.5 stone down to about 9.*	Psychological effects	Escapism
			Relaxation
			Happiness
			Alertness/Energy
		Physical effects	Weight loss
		Preference	Strength
			Affordability
			Curiosity
			Boredom
			Duration
P_6_	*More involved in the music, film … my playing sounded great … [it] wasn’t very great … they didn’t have any downside at all … they didn’t relax you or send you to sleep–quite the opposite … I went back because of the power it had over me. It scared me and I wanted it to … I wanted to tame it … I wanted to be able to do it without freaking out … it would last for ages because it’s so strong*	Psychological effects	Alertness/Energy
			Improved subjective experiences
			Overcome and control previous negative experiences
		Preference	Strength
			Duration
P_7_	*You just get more for your money worth [than “street” amphetamine]*	Preference	Quality
			Affordability
P_8_	*I like the relaxing, calming numb feeling … The effect, it’s quicker than marijuana. You get higher quicker … faster*	Psychological effects	Relaxation
		Preference	Onset of action
P_9_	*It was quite stimulating … had an overriding desire to argue with Christians … improved my darts. It deepened my appreciation of Johnny Cash … You legalise [it] and I’ll be back on it …*	Psychological effects	Alertness/Energy
			Improved subjective experiences
		Preference	Legal status
P_10_	*Make myself happy … made me stronger … gives me confidence … I eat every day [resulting in weight gain when previously underweight]*	Psychological effects	Happiness
			Confidence
		Physical effects	Weight gain
P_11_	*Totally a good feeling basically, made me feel quite happy and cheerful … I tried most of them really to see what they were like I stuck to the illegal ones cos they work better … I thought, yeah I’ll try that–see what happens*	Psychological effects	Happiness
		Preference	Quality
			Curiosity
P_12_	*[Stimulant NPS] gives me more energy … [cannabinoid NPS] calms me down … It enhances–changes your perspective on stuff … Helps me listen to music … I’ve lost a lot of weight doing them. I was 18 and a half stone before I started doing them … I like to keep the weight off … I’ve got a bad leg and it takes that pain away*	Psychological effects	Relaxation
			Alertness/Energy
			Improved subjective experiences
		Physical effects	Weight loss
			Analgesia

4. Negative experiences associated with NPS use

**Table 4:** Negative experiences associated with NPS use. A list of all the themes and sub-themes, with respective quotes by participants.

Participant	Quote	Theme	Sub-Theme
P_1_	*My lip and my front teeth just went numb … it makes your eyes water … and your nose burnt sometimes. The nostrils would burn [when snorting NPS]*	Physical effects	Administration site
P_2_	*A little bit dearer [than “street drugs”] … It has turned me into a more devious person as well, although I’ve never stolen … I am the nastiest person on it … I don’t like the shakes … it makes me itch a bit … eczema … it’s a really dangerous sweat, it’s pretty embarrassing*	Psychological effects	Personality changes
		Physical effects	Tremor
			Eczema
			Sweating
			Itching
		Preference	Affordability
P_3_	*I’ve had thoughts of … killing myself … not wanting to be here … I didn’t like the effects … it’ll kill you … the heartbeat on the high ones … you feel like, I’ve just injected here and my heart’s going. It feels like I’m dying … That’s a vein there and it’s just left a massive lump … It’s too powerful*	Psychological effects	Suicidal ideation
		Physical effects	Administration site
			Cardiac
		Preference	Strength
P_4_	*I couldn’t go out, struggled concentrating … They don’t make you feel good; all they do is make your heart feel like you’re having a heart attack … heart fluttering, palpitations … Too strong, over-powering*	Psychological effects	Concentration
			Impaired activity of daily living
		Physical effects	Cardiac
		Preference	Strength
P_5_	*I stopped cleaning my flat … go for about 4 days without eating … lost interest in my home. I wasn’t paying my bills … washing up not done for months … got sacked … all I wanted to do was get money and buy legal highs. That was my whole life … crawling around my carpet on my hands and knees … try and get enough for another rollie … that’s how addictive they were … Eventually the police turned up and I basically got detained under the Mental Health Act for 24 h, until the effect of the drugs wore off … I started fitting in the street and I just couldn’t control my body … it no longer had that kind of kick to it … I stopped buying it … you get a variation … the illegal high stuff is more consistent in its strength*	Legal	Detained (Mental Health Act)
		Psychological effects	Impaired activity of daily living
			Self-neglect
			Loss of control
			Dependency/Addiction
		Physical effects	Seizures
		Preference	Strength
			Quality
P_6_	*Too potent … I hate the fact that this is controlling me … I don’t have control … Couldn’t remember what I was doing … completely confused … Gave me panic attacks … Wouldn’t have been in any fit state to go out … It seemed to speed my heart up to a frightening degree … I really thought I was gonna have a heart attack … my heart was thumping away madly … Hundreds of times stronger … it’s easy to go over-board … the intensity of it was just on another level … I think they’re more dangerous because they are just so intense … It could be a little bit hit and miss … it wouldn’t have the same sort of effect*	Psychological effects	Impaired activity of daily living
			Confusion
			Impaired memory
			Panic attacks
			Loss of control
		Physical effects	Cardiac
		Preference	Strength
			Quality
P_7_	*It was pretty strong and I thought I was gonna collapse and die … It was the strength of it*	Preference	Strength
P_8_	*I want to keep to that high all the time … sleep [to stop] … It might make you cough … sweats*	Psychological effects	Dependency/Addiction
		Physical effects	Coughing
			Sweating
P_9_	*Abscesses*	Physical effects	Administration site
P_10_	*You get more addicted. You wanna another bag, another bag and another*	Psychological effects	Dependency/Addiction
			Cravings
P_11_	*I stuck to the illegal ones cos they work better*	Preference	Quality
P_12_	*I need them, cos I’ve been doing them for 6–7 years … I don’t like having to wake up when I haven’t got the money for them. Then I have to try to get the money for them. I don’t like that part of it … they’re quite addictive … done some quite reckless stuff to get them … trouble with the police … Makes me shake a lot … all the time*	Psychological effects	Dependency/Addiction
		Physical effects	Tremor
		Legal	Police involvement

## 4. Discussion

This study contributes to existing knowledge of how NPS are used by individuals, who engage with specialist adult SMSs. Seven (58%) reported opiates (illicit heroin/morphine) as their primary substance of use; this is in keeping with the finding that five (42%) of participants were in receipt of opiate substitution prescriptions. For a quarter of individuals, NPS was their primary substance of use in accordance with PHE classification [15], suggesting that while not as common as opiates (which SMSs are more familiar with treating [15]), NPS use is prevalent among individuals accessing treatment services, as previously suggested by both national and international findings [7,8,39].

The interviews allowed individuals to describe their experiences with all types and routes of administration of NPS; therefore, adding to the existing evidence base, particularly since previous studies [24,40] have largely focused on stimulant NPS, which were injected. Audio recordings allowed for more complete data and detailed transcriptions than relying on memory or notes alone and piloting should have increased the likelihood of individuals sharing their personal views and experiences [42,43,44].

Harm reduction measures aim to reduce the harm that someone may experience because of their ongoing substance use [45,46]. These include but are not limited to avoiding poly-pharmacy, administering by routes associated with less risk (such as smoking rather than injecting), not sharing drug paraphernalia and using smaller amounts. This study, as with other research, found that individuals did not usually know exactly which chemical they were taking [19,50]. Amount of experience with NPS and frequency of use varied greatly, from very occasional up to every 20 min. Frequent use was mainly observed with synthetic cannabinoid receptor agonists (SCRAs) and stimulant NPSs. Due the wide chemical diversity of SCRAs, little is known on their pharmacokinetics [51]. However, stimulants such as mephedrone may have a short duration of action warranting repeated dosing [52]. Overall substance use patterns fluctuated over time (with varying self-perceptions), which may be explained by individuals being at different stages in their recovery journeys. Participant ages ranged from 26 to 59 (SD = 9); other studies have found and that most people using NPS are under 30 years old [1]. The difference may be because this study unlike national data reflects the age of those requiring treatment. Our results therefore show that SMSs need to manage individuals with a range of ages presenting with NPS use.

Previous studies and national reports have outlined a complex relationship between NPS and other substances, and the need for further investigation. Factors affecting use include substance availability, effects, safety profile, quality, price, purity and legal status [1,16,21,22,23,38]. This study equally found that NPS were favored over other substances for their perceived legal status, improved price, availability and higher quality, including quicker onset and duration of action, therefore adding to the existing knowledge base.

The large proportion (*n* = 9; 75%) of participants who stated they had experience with SCRAs is perhaps to be expected (SI2), given that cannabis, the traditional illicit substance, which these substances are attempting to mimic, has the highest prevalence rate of all illicit substances [53]. Preference for NPS type appeared to be dependent upon the required effect (usually stimulating or sedating). Some wanted to gain confidence, energy and alertness; others wanted to “escape reality”, experience euphoria, feel happy, relaxed or were potentially self-medicating for underlying psychological trauma (and in one case (P_12_) for leg pain). This has been found in other studies [30,54], where stimulating effects were particularly popular [21,31,54]. One study found that psychedelic effects were commonly favored [30]; although this could be due to the differing study environments (such as dance venues and festivals). Individuals also reported using NPS to alleviate feelings of boredom and out of curiosity, which has also been found previously [1,21,55] and similarly for traditional illicit substance use [1].

NPS were often mixed with other substances [34,50] and administered by a variety of routes, although snorting/injecting stimulant NPS and smoking SCRAs, the latter in combination with tobacco (*n* = 4; 33%) predominated (SI2). Stimulant and SCRA NPS were also used sequentially to balance the effects of each other. Excessive binging for long durations on stimulant NPS, followed by substances with sedating and relaxing effects to “come down” supports the results of another study [24]. Poly-pharmacy is associated with an increased risk of drug interactions and side-effects including overdose and death [7,45], so harm reduction advice, which SMSs offer, should include: overdose awareness, how to reduce frequency, avoid “binges”, minimize polysubstance use, manage altered tolerance levels as well as the provision of take home naloxone [24,56].

The amount participants spent on their NPS varied widely and was affected by how much money they had access to, the source of NPS supply and pattern of use. To reduce costs NPS were bought in bulk or smoked with tobacco and one person (P_3_) also described dealing NPS to fund his habit. NPS were described as comparatively cheap to traditional illicit drugs which has been similarly suggested by the Home Office [1]. A Dutch study has found them to be similar in price to the substances they are attempting to mimic [30] and this may be accounted for by differences between England and Holland drug markets.

Strength of NPS was perceived as both a positive and negative. Some found them to be too strong, leading to unwanted effects and overdoses; others preferred them for their strength, considering them to be more cost-effective and of higher quality. Overall, NPS use reduced if strength and quality was variable, and the NPS was viewed as inferior rather than superior to traditional illicit drugs; this may be highly dependent on the local drug markets and has been similarly identified by the Home Office [1]. 

Our findings support other research, which identified that the rapid proliferation of NPS use may be changing drug taking habits [22]. Several individuals reported NPS alleviating withdrawal symptoms including cravings, from other substances and in some cases completely substituted with NPS. SMSs should therefore provide individuals with advice on how to manage withdrawals (from all substances). Using NPS in addition to traditional illicit drugs or prescribed substitute medication to potentiate effects was also reported, particularly when then the person was presenting as more chaotic as found in similar studies [24]. This is unsurprising since individuals in the early stages of recovery commonly present with more complex and riskier patterns of substance use.

Participants reported obtaining NPS from various sources; the majority had experience of using headshops (*n* = 9; 75%) (SI2) in contrast to a qualitative study that found that NPS were usually obtained from friends or online [30]. Headshops were often preferred for their convenience (particularly if they could avoid “dealers”), with usage reducing when they could no longer be legally sold. However, legal status did not always deter use, supporting Home Office findings, which suggest this may be due to the impact of legal status upon the quality of NPS [1,3]. Previous studies have equally conflicted in their findings on the effect of legal status on the decision to use NPS [24,30]. Purchasing them from high street vendors may add to the perception of this being “normal” behavior and the risk of them being considered as “safe” and “legal” and consequently increase NPS use. For example, one person (P_3_) felt it was permissible to sell them because they did not consider them to be “drugs” in the same way as traditional illicit substances. Recent studies showed that there is an underlying competition between cryptomarkets and street networking, which may drive high quality of illicit substances as well as NPS [25,26,27,28]. However, an international drug testing service that was offered to cryptomarket users suggested that this may not be the case [25].

Participants described a wide range of physical and psychological problems including symptoms of NPS dependency reflecting national and international findings [4,8,57,58]. Similar to the findings of other studies [31,57,58], reported physical health effects included tremor, coughing, itching, seizures, eczema and sweating, and problems associated with specific routes of administration such as venous abscesses. Like other studies, cardiac effects were reported (*n* = 3; 25%) (SI2), usually following stimulant NPS use and sometimes lasting a few days [31,57,58]. While individuals described some of these effects as sometimes being prolonged, the long-term effects remain unknown. Individuals were not specifically asked about their experiences of acute compared to chronic effects or to distinguish between side-effects and withdrawal symptoms. This suggests further study development and provides additional support for the need for longitudinal research [1]. Unpleasant psychological effects, included feelings of loss of control, difficulties concentrating, impaired memory, confusion, personality changes, panic attacks and cravings; sometimes leading to crime, impaired activities of daily living and self-neglect. Suicidal ideation and in one case (P_5_), being sectioned under the Mental Health Act following prolonged NPS use was reported. Individuals with pre-existing mental health conditions (“dual diagnosis”) may be particularly at risk of psychological problems and local integrated pathways should outline how to obtain the required support quickly and effectively, especially in acute situations such as the person disclosing suicidal ideation.

High risk administration practices and side-effects such as vein damage as a direct result of the route of administration were described, although not as significant as identified by a similar Irish study, perhaps because it only included NPS mainly administered by injection [24]. To ameliorate this, individuals actively undertook harm reduction approaches such as switching from injecting to snorting and using less [45]. It is important that SMSs appropriately signpost to additional sources of help and support, for example tissue bioavailability services.

### 4.1. Limitations of the Study

Repeat interviews and member-checking was not implemented due to difficulties in re-establishing contact with the participants, time constraints and geographical problems. The purposive sampling [42,43,44] approach enabled suitable participants to be identified. Although small, tentative data saturation was achieved, as has been found to occur in other research [24,42,43,44,49]. A larger sample size may have enabled more generalizability; however, this smaller sample size allowed for more in-depth analysis.

Interviews require people to discuss their experiences in an artificial environment, which may lead to problems with reliability. However, this approach of using semi-structured interviews to obtain detailed information from individuals regarding their NPS use has been successfully used previously [23,24,30].

Data was collected in a predominantly rural environment and further research should be conducted in more urban areas. The Devon RISE and Addaction Cornwall SMSs were almost equally represented (7:5 respectively). Men dominate SMSs and reflecting this, all our participants were men [1,7]. With a larger sample size, women may have been included, providing the opportunity for comparison.

Data was collected in 2015, when many NPS were sold openly in headshops and NPS markets have since changed. Currently, under the UK NPS Act 2016, headshops can no longer legally sell NPS and a clear shift from the surface to the dark net was observed [59] and motivation for NPS use may have consequently evolved. Implications of the new legislation include intentional poor disclosure of NPS use to HCPs and NPS emerging on the illicit market, often sold as the traditional illicit drug the NPS mimics, to increase profit margins [55]. The delayed reporting of this study is due to the time needed for the necessary approval for publication 

### 4.2. Implications for HCPs and Policy-Makers

The way that the participants described NPS, easily made them identifiable in accordance with PHE categories used by NDTMS, therefore providing support for this approach in clinical practice [15]. However, there were significant discrepancies between NPS use recorded on Halo for the purposes of NDTMS [16] and disclosures made by individuals during the interviews. Therefore, all individuals presenting to SMSs should be asked about their use of NPS, the name of the substance, and reassessed regularly, because they may not routinely present or perceive this as an issue, especially if other substances dominate their pattern of substance use or if it changes over time. Additionally, this study highlights that individuals should be asked about NPS regardless of their age. With the increasing emergence of NPS (from approximately 478 in 2015 when this data was collected [14] to 740 in 2016 [11]), increasing chemical diversity, potency, and changing degree of purity [14,25], it is important that HCPs reassess regularly the use of novel or existing NPS and tailor harm reduction approaches to individuals. The importance of offering harm reduction interventions was demonstrated by the positive impact that they had on individuals; however, the reports of managing withdrawal symptoms by diverting prescribed medication or otherwise using polypharmacy must not be underestimated in clinical practice. HCPs must check that prescribed doses are optimized to reduce cravings and “on top” use. Additionally, adherence to medication regimens must be checked and community pharmacy teams must promptly notify prescribing services of missed doses.

Consequently, treatment needs may also fluctuate over time, so SMSs need to be able to accommodate this and regularly review the person’s progress. These findings also provide support for using interviews to elicit more accurate data regarding people’s NPS use and highlights that reviewing NDTMS data in isolation to assess the extent of NPS use is inadequate. This may be for a variety of reasons, including lack of staff vigilance when completing the required data set and suggests a potential training need.

Because of their overall experiences, some strongly felt that they would never use NPS again, while others continued to use despite their negative consequences. These findings corroborated with previous research on SCRAs, where users reported that these substances completely “hijacked their personalities” [60]. These negative effects may have a significant impact upon the person, including their employment, housing status and criminal record, and the wider community. SMSs are traditionally skilled in providing support with such problems but promoting awareness among staff about how best to identify individuals requiring more intensive support, or those whose needs may need to be prioritized, such as individuals with pre-existing conditions, those who are homeless or subject to CTOs, may enable this to be provided more promptly.

There was evidence that the SMSs were raising awareness of harm reduction by advising on alternative routes of administration, such as switching from injecting to snorting and offered safer injecting technique sessions. Risks may be further reduced if the substance could be tested to confirm its content prior to administration and harm reduction advice [5,61] was provided at the time of purchase. Currently, neither is currently permissible in UK SMSs, therefore adding further weight to reviewing existing legislation.

SMSs need to understand the motivation for using NPS: when individuals disclosed their substance use histories, they were highly variable. In one case, iatrogenic dependency (P_1_) was described, though reasons for use were frequently associated with traumatic life events, often from childhood. This has been found to occur in other NPS studies [21] and SMSs should therefore be sensitive to the needs of these individuals and tailor psychological interventions accordingly. When more significant disclosures are made, other services such as bereavement counselling and mental health teams should be signposted so that any unresolved issues can be managed effectively.

Some described improved abilities and experiences with NPS, often acknowledging this was perceived because of the mind-altering effects of the substance, such as having energy while on a night out and enjoyment of activities such as playing music. Such information may be of interest to policy-makers, where psychoactivity of newly emerging NPS has not yet been demonstrated. One individual (P_6_), reported a desire to use NPS to overcome their previous negative experiences with these substances, alongside entrenched addictive behaviors and boredom due to a lack of meaningful daytime activities. This highlights the need for SMSs to promote a variety of psychosocial interventions to help occupy an individual’s time in substance-free environments, which are tailored to the person’s needs to enable them to progress with their recovery. Supporting people to develop skills which will enable them to find work is important because the number of participants in employment was comparatively low and boredom was cited as a reason for NPS use, which may be compounded by rural locations. Some may require more intensive RC support and there should be adequate provision for this. 

The main positive physical health effects reported were upon weight: three (25%) stated that using predominantly stimulant NPS to keep their weight down was one of their main motivators for use. This may be a particular problem once the person stabilizes their NPS use and weight gain is observed, which may result in the occurrence of other eating disorder symptoms. In contrast, one individual (P_10_) reported appetite stimulating effects which they found to be beneficial as they were otherwise losing weight due to self-neglect because of their chaotic drug use. However, it cannot be assumed that the food they were selecting to eat included “healthy options”. In these situations, individuals should be offered supportive explanations that if their substance use stabilizes their weight will usually return without the need for stimulants. Malnutrition may be common in people in substance use treatment, which is one of many reasons why SMSs supporting activities such as “breakfast clubs” and the provision of “life-skills” courses which incorporate cooking techniques and general nutritional advice, should be actively encouraged. Everyone involved in the care of people with substance use problems (including GPs and community pharmacists in addition to specialist SMSs) should be vigilant for eating disorder behaviors and provide the required support or signpost as required: there may be training needs.

Three participants were subject to CTOs because of their substance use and would therefore require regular drug testing for SMSs to report on their progress. Since NPS may not always be detectable in routine drug screens and can produce false positives [5,55,61], SMSs could consider more specialist NPS-specific tests. These may be expensive (and not always possible due to the novelty of the substances), but if used sparingly and appropriately, they may be a useful tool. However, this is not without risk, since it may also perpetuate the demand for “new” NPS that are not detectable. Half of the participants were in receipt of a prescription for oral opiate substitution treatment and using NPS as a replacement for such medication was reported. The diversion of prescribed medication is highly likely in such situations. Therefore NPS-specific tests may also be useful for supporting decisions around continued prescribing in circumstances where there is evidence of “on top” use or diversion is suspected. Staff should receive appropriate training so that they are vigilant for “on-top” use, diversion and substitution of the person’s prescribed intervention with NPS, particularly if the individual is under a CTO.

NPS treatment systems are complex and require structured partnerships between multidisciplinary teams with a broad range of competencies. This includes: A&E departments, sexual, social care and mental health services, prison, probation staff and Young Offender teams, community adult and young people SMSs, youth services and organizations such as the police [6]. This is required to manage the myriad of health, social and criminal problems associated with NPS use and to address the diverse needs of individuals including young people, MSM, prisoners, and the homeless population. This approach should also involve people who use NPS and their carers. Drug detection is key to support diagnosis and treatment planning, adherence and outcomes [5,6,55,61]. Proactive, engaging and competent SMSs are needed to respond appropriately to meet the needs associated with NPS use. Regular education and training is of paramount importance to inform about types and degrees of harm, new patterns and trends of recreational drug use and changes to the drug scene [6]. NPS use should be considered in clinical assessments and management plans should be devised accordingly [20].

## 5. Conclusions

This study found that NPS are used by individuals across a range of ages, presenting to SMSs in Devon and Cornwall, but is highly variable. NPS may be favored over other substances for their perceived legal status, price, strength, availability and better quality, including quicker onset and duration of action and improved side-effect profile. Consequently, NPS use may be dependent on local drug markets and reduce if perceived to be variable in strength and quality in comparison to traditional illicit drugs. SCRAs and stimulant NPS are most frequently used and preference for NPS type is often dependent upon the required effect. Using poly-pharmacy (including NPS, traditional illicit substances and prescribed medication) to potentiate effects, manage side-effects and withdrawal symptoms occurs, sometimes resulting in displacement of substances. Therefore, SMS must remain vigilant and consider the use of NPS-specific tests, especially when substitute medication is prescribed. NPS are frequently administered by a variety of routes, including high risk injecting practices, though individuals can respond well to harm reduction interventions, which should be routinely provided and include the management of withdrawals. Results showed that a wide range of problematic physical and mental health effects may occur, including symptoms of dependency, which may lead to criminal activity, impaired activities of daily living and self-neglect. People’s perception of the severity of their use, frequency and amount used varies widely and may change depending on their recovery journey, consequently fluctuating for individuals over time. People use NPS for a variety of reasons, including stimulant types taken for the intention of losing weight. As malnourishment is often an issue for individuals accessing SMS, the multidisciplinary team must be attentive, frequently revisit the current pattern of substance use and any associated issues, promptly refer to other services accordingly and may require further training. Local integrated pathways for individuals presenting with more complex issues and particular needs such as dual diagnosis and tissue bioavailability should be established to enable prompt access. SMSs should engage individuals with psychosocial interventions and meaningful daily activities, especially in more rural areas. It is important that current approaches to existing treatment strategies are adapted and recovery plans tailored to an individual’s needs. Education and training is needed for HCPs working within various services where people who use NPS are encountered. Multidisciplinary and multiagency approaches should be nationally adopted to capture changing trends of NPS use.

## Figures and Tables

**Table 1:** Summary of information obtained from the electronic record system (Halo for all 12 participants.

Participant	P_1_	P_2_	P_3_	P_4_	P_5_	P_6_	P_7_	P_8_	P_9_	P_10_	P_11_	P_12_
Age (years)	28	33	34	31	40	37	36	59	45	44	26	32
Sex	Male	Male	Male	Male	Male	Male	Male	Male	Male	Male	Male	Male
Service attended	RISE Devon	RISE Devon	Addaction Cornwall	Addaction Cornwall	RISE Devon	Addaction Cornwall	RISE Devon	RISE Devon	RISE Devon	Addaction Cornwall	Addaction Cornwall	RISE Devon
Under Criminal Justice	No	No	Yes	No	No	No	No	No	No	No	Yes	Yes
Housing problem	None	None	None	None	None	None	Acute problem	None	None	None	None	None
Employed	Yes	No	No	No	Yes	No	No	No	No	No	No	No
Substitute prescription	Methadone oral liquid 40 mg/day	None	Buprenorphine sublingual tablet 8 mg/day	Methadone oral liquid 50 mg/day	None	Methadone oral liquid 30 mg/day	None	None	Methadone oral liquid 100 mg/day	None	None	None
Primary Drug *	Morphine	Cocaine	Heroin	Heroin	NPS-stimulant	Heroin	Heroin	NPS-Cannabis	Heroin	Alcohol	Heroin	NPS-other
Secondary Drug *	Codeine	NPS–stimulant	Cannabis	None	NPS-Cannabis	None	Diazepam	None	None	None	Cannabis	None
Tertiary Drug *	None	None	None	None	None	None	NPS-other	None	None	None	None	None
Injecting status (number of days injected in last 28 days)	0	0	28	0	0	8	0	0	23	0	12	0

* In accordance with PHE classification [15] recorded for NDTMS purposes [16] on Halo.

## Supplementary Materials

The following are available online at http://www.mdpi.com/2076-3425/8/4/58/s1. Supplementary Information 1 (SI1): Semi-structured Interview Guide; Supplementary Information 2 (SI2): Summary of Themes and Sub-themes
