# Supplementary material for: Exploration of the Use of New Psychoactive Substances by Individuals in Treatment for Substance Misuse in the UK
Source: Brain Sci. 2018 Mar 30;8(4):58. doi: 10.3390/brainsci8040058 (PMC5924394; doi:10.3390/brainsci8040058)
Supplement: Supplementary file 1 [file brainsci-08-00058-s001.pdf]

Supplementary Information 1 (SI1)

Semi-structured Interview Guide

<u>Opening statement:</u>

"Thank you for agreeing to take part in this interview. Before we start I need to make sure you're aware that everything that we discuss is confidential. I will be recording our conversation and may need to make some notes but this will be kept secure and separate from any information that could identify you. If I use any quotes from our discussion they will not identify you directly. If you tell me any information that makes me concerned that someone may be likely to suffer harm I may need to look into this further and I may need to break confidentiality to protect them. Before we start do you have any questions or concerns?"

"So we'll make a start: if you have any questions or change your mind about speaking to me as we go along then please let me know."

<u>Questions:</u>

"I would like to find out more about the use of NPS amongst the people who are currently using our service so will be asking you some questions around this theme."

(Explain NPS definition if needed)

Explore history of substance misuse (5mins):

- "What substances (e.g. heroin, cannabis, alcohol) do you have experience of using and how/when do you use them?
- Have you taken NPS before?"

Explore type and pattern of use of NPS that have been taken before, (10mins):

- "What types of NPS have you used? (e.g. cannabinoids, speedy ones)
- How did you take them? (e.g. smoke them, swallow pills, skin-popping)
- How did they fit in with your other substance misuse?
- Where did you get them from? (e.g. headshop, online, friend, relative, dealer)
- How often did you use them? (e.g. every day, a couple of times a week)

Explore positive/negative experiences of NPS use (10mins):

- "What did you like about them?
- Is there anything you didn't like about them?
- How did they affect your mental health?
- Have they affected your physical health?"

<u>Closing statement:</u>

"Is there anything that you would like to add or talk about that we haven't covered?"

"Do you have any questions?"

"Thank you for your time."

SI2: *Summary of Themes and Sub-themes*

Table SI2: Summary of number of participants who mentioned each of the themes and sub-themes.

	Theme	Sub-theme	Number of participants
Type and pattern of NPS use	NPS type	Cannabinoid ONLY	1
		Stimulating ONLY	1
		'Other' (not otherwise specified) ONLY	1
		Stimulating & Cannabinoid	7
		Stimulating, Dissociative & Hallucinogenic	1
		Stimulating, Cannabinoid & Sedating	1
	Source	Friend ONLY	1
		Online ONLY	1
		Headshop ONLY	6
		Headshop & Online	1
		Headshop, Friend, Online & Other (newsagent)	1
		'Street dealer' & Friend	1
		'Street dealer' & Headshop	1
	Frequency	Occasional	3
		Daily (several times)	5
		Binging	2
	Route of administration	Smoking ONLY	3
		Nasal ONLY	1
		Intra-venous injection ONLY	1
		Smoking & Intra-venous Injection	2
		Nasal & Intra-venous injection	1
		Oral, Nasal & Smoking	2
		Nasal, Smoking & Intra-venous Injection	2
	Concomitant use	Cannabinoid NPS to end stimulant NPS use	2
		Opiates to end stimulant NPS use	1
		Benzodiazepines to end NPS use	1
		Cannabinoids with tobacco	4
		Potentiating effects	1
		Displacement (prescribed opiates)	1
		Displacement (illicit opiates)	2
		Displacement (alcohol)	1
	Preference	Accessibility	4
		Avoid dealers	2
	Harm reduction	Changed route of administration	1
		Safer injecting training	1
		Changed dose	1
	Affordability	Budgeting	2
		Cheap	1
		Dealing	1
		Bulk-buying	1
		Cannabinoids with tobacco	1
Positive experienc	Psychological effects	Escapism	3
		Relaxation	4
		Happiness	4
		Euphoria	1

		Confidence	2
		Alertness/Energy	5
		Improved subjective experiences	3
		Overcome and control previous negative experiences	1
	Physical effects	Weight loss	3
		Weight gain	1
		Analgesia	1
	Preference	Strength	3
		Quality	3
		Legal status	2
		Affordability	3
		Curiosity	2
		Boredom	1
		Duration	2
		Onset of action	1
Negative experiences associated with NPS use	Physical effects	Administration site	3
		Tremor	2
		Eczema	1
		Sweating	2
		Itching	1
		Cardiac	3
		Coughing	1
		Seizures	1
	Psychological effects	Personality changes	1
		Suicidal ideation	1
		Concentration	1
		Dependency/Addiction	4
		Cravings	1
		Impaired activity of daily living	3
		Self-neglect	1
		Loss of control	2
		Confusion	1
		Impaired memory	1
		Panic attacks	1
	Preference	Affordability	1
		Strength	5
		Quality	3
	Legal	Police involvement	1
		Detained (Mental Health Act)	1
